# The Human NADPH Oxidase, Nox4, Regulates Cytoskeletal Organization in Two Cancer Cell Lines, HepG2 and SH-SY5Y

**DOI:** 10.3389/fonc.2017.00111

**Published:** 2017-05-31

**Authors:** Simon Auer, Mark Rinnerthaler, Johannes Bischof, Maria Karolin Streubel, Hannelore Breitenbach-Koller, Roland Geisberger, Elmar Aigner, Janne Cadamuro, Klaus Richter, Mentor Sopjani, Elisabeth Haschke-Becher, Thomas Klaus Felder, Michael Breitenbach

**Affiliations:** ^1^Department of Laboratory Medicine, Paracelsus Medical University, Salzburg, Austria; ^2^Department of Cell Biology, Division of Genetics, University of Salzburg, Salzburg, Austria; ^3^Department of Internal Medicine III with Hematology, Medical Oncology, Hemostaseology, Infectious Diseases, Rheumatology, Oncologic Center, Laboratory for Immunological and Molecular Cancer Research, Paracelsus Medical University, Salzburg, Austria; ^4^First Department of Medicine, Paracelsus Medical University, Salzburg, Austria; ^5^Obesity Research Unit, Paracelsus Medical University, Salzburg, Austria; ^6^Faculty of Medicine of the University of Prishtina, Prishtina, Kosovo

**Keywords:** NADPH oxidase, hydrogen peroxide, signaling, actin cytoskeleton, cell migration

## Abstract

NADPH oxidases of human cells are not only functional in defense against invading microorganisms and for oxidative reactions needed for specialized biosynthetic pathways but also during the past few years have been established as signaling modules. It has been shown that human Nox4 is expressed in most somatic cell types and produces hydrogen peroxide, which signals to remodel the actin cytoskeleton. This correlates well with the function of Yno1, the only NADPH oxidase of yeast cells. Using two established tumor cell lines, which are derived from hepatic and neuroblastoma tumors, respectively, we are showing here that in both tumor models Nox4 is expressed in the ER (like the yeast NADPH oxidase), where according to published literature, it produces hydrogen peroxide. Reducing this biochemical activity by downregulating Nox4 transcription leads to loss of F-actin stress fibers. This phenotype is reversible by adding hydrogen peroxide to the cells. The effect of the Nox4 silencer RNA is specific for this gene as it does not influence the expression of Nox2. In the case of the SH-SY5Y neuronal cell line, Nox4 inhibition leads to loss of cell mobility as measured in scratch assays. We propose that inhibition of Nox4 (which is known to be strongly expressed in many tumors) could be studied as a new target for cancer treatment, in particular for inhibition of metastasis.

## Introduction

In an article that appeared in 2012, we showed that the monocellular yeast, *Saccharomyces cerevisiae*, contains a genuine NADPH oxidase, Yno1, and provided evidence for a function of this enzyme in regulation of the actin cytoskeleton of the yeast cell (1). We now wanted to extend this finding by studying the human NADPH oxidase Nox4, which is involved in the pathomechanism of human cancer cells. Regulation of the assembly and polarity of the actin cytoskeleton of human cells is required for all steps of tumor development and in particular is prerequisite for cell mobility and, therefore, for metastasis of human cancer cells. This fact, and the other unique properties of Nox4 listed subsequently prompted us to choose Nox4 among the group of seven NADPH oxidase (Nox) isoenzymes expressed in human cells.

Nox4 displays the largest sequence identity and similarity of all human Nox enzymes to the yeast NADPH oxidase, Yno1. Direct pairwise sequence comparisons (2) of Yno1 with all seven human Nox enzymes shows that Nox4 is the best match for Yno1. In a sequence window of 553 amino acids, the two NADPH oxidase sequences share 29.3% identity and 50% similarity. For comparison, the second best sequence match is Nox5. In a window of 565 amino acids, Yno1 and Nox5 share 23% identity and 42% sequence similarity.

Nox4 is different from all other human Nox enzymes because it is not associated with exactly the same group of regulatory proteins which is well studied in the case of Nox2, like p47phox and p67phox. However, p22phox is required for human Nox4 expression (3, 4) and a newly discovered p22phox binding partner, Poldip2, seems to be necessary for Nox4 activation (5). Nox4 is expressed constitutively in many different cell types (6). Nox4 exists as a number of splice variants in human cells. However, the significance of the splice variants for tumor biology is not known in detail. One of the splice variants (Nox4D) lacks all transmembrane helices, is located in the nucleus and highly expressed in vascular endothelial cells (7). Four other splice variants are apparently dominant negative mutant forms lacking the NADPH and/or FADH binding sites (8, 9) with no known direct relevance for cancer growth. The K_M_ of Nox4 for oxygen is unusually high (on the order of the actual oxygen partial pressure in tissue) indicating that Nox4 might be a relevant oxygen sensor for human cells (10).

Nox4 was found to be expressed in several tested cancers and cancer cells lines (6). Inhibition experiments with siRNA constructs showed that Nox4 is specifically needed for metastasis and also for epithelial to mesenchymal transition (EMT), a process needed for invasiveness of tumor cells mediated by regulating the actin cytoskeleton (10, 11). Details of the mechanism by which Nox4 is involved in regulation of the action cytoskeleton, and therefore in the EMT and in migration and metastasis of tumor cells largely are unknown, however, evidence was published recently that the signaling pathway in which Nox4 is embedded could be the TIS21-PI3K-Akt1 pathway (12, 13).

Nox4 is the only human Nox, which was found to be located in the ER, as shown in the present and in a previous article (14). In the two cancer cell lines used by us, Nox4 was exclusively seen in the ER (see Results and Discussion section). In some of the other cells tested, location in the nuclear membrane, in the plasma membrane and in mitochondria was also found (6, 15). Details about the correlation of subcellular location with function or about relocation from ER to nucleus, etc. are not yet available.

The product of the Nox4 catalyzed reaction, H_2_O_2_, is assumed to be formed in the lumen of the ER (6), where no known SOD is present. Nox4, but none of the other human Nox enzymes, produces H_2_O_2_ directly (without help from superoxide dismutase) via superoxide (10). Measurements of both H_2_O_2_ and superoxide *in vitro* showed that about 85% of the oxygen is converted to H_2_O_2_ while only 15% is converted to superoxide. It is unknown presently whether the yeast enzyme, Yno1, can produce H_2_O_2_ directly from oxygen (1).

It is shown by a host of medical papers that the H_2_O_2_ produced acts as a second messenger molecule in human cells, promoting cells at several stages of the life history of tumor formation, starting from immortalization (loss of cell cycle arrest), to the EMT, tumor angiogenesis, activation of HIF1alpha leading to a hypoxia-like metabolic transition of the cells, and finally to invasiveness and the potential to metastasize (13, 16–18). Examples for signaling modules that were shown to take part in Nox4 signaling in different cell types are TGF-β and phosphotyrosine phosphatase 2B (6). Paradoxically, Nox4 expression is not only needed for proliferation but also for apoptosis of tumor cells. Pancreatic tumor cell lines became resistant to apoptotic stimuli when Nox4 was silenced by RNAi [(19) summarized in Ref. (6)]. In part, the induction of apoptosis uses the same signaling pathways as proliferation (for instance, TGF-β). This apparent contradiction is presumably resolved by considering the combinatorial nature of signaling modules and the gene expression differences in the tumor and primary non-tumor cell lines used for these experiments. These facts must be given careful consideration in the development of Nox4 as a target for cancer therapy.

A large number of pharmacological inhibitors of the human Nox enzymes have been studied (20, 21), but none of them is specific for Nox4 and very little is known about their mechanisms of action and side reactions. Relatively recently, promising natural compounds (still not specific for Nox4) were tested for their therapeutic action *in vivo* and their biochemical action *in vitro* (20).

In the present article, we present evidence that Nox4, similar to its yeast homolog, creates a ROS signal leading to re-structuring of the actin cytoskeleton in two human tumor cell lines. Inhibition of Nox4 leads to a loss of cell mobility which is pictured by changing the polarity of the actin cytoskeleton and prohibits cell migration *in vitro*. Therefore, it is encouraging to block Nox4 pharmacologically as a means to counteract the metastatic potential of cancer cells.

## Materials and Methods

### Cell Culture and Transfections

HepG2 cells were grown in MEM media (ThermoScientific) supplemented with 10% fetal bovine serum (FBS; Life Technologies/Gibco), 2 mM l-glutamine (Sigma-Aldrich), 1 mM sodium pyruvate (Sigma-Aldrich), 1× non-essential amino acids (Sigma-Aldrich), and 100 µg/mL gentamycin (ThermoScientific). SH-SY5Y cells obtained from ATCC were cultured in Dulbeco’s Minimum Essential Medium (DMEM)/F12 (1:1 mixture) supplemented with 10% FBS, 2 mM l-glutamine, 1 mM sodium pyruvate, 1× non-essential amino acids, and 100 µg/mL gentamycin. Medium was changed every 2–3 days, and cells were subcultured at a density of 70–80%. For siRNA-mediated Nox4 knockdown, 7.5 pmol of Nox4-specific silencer RNA or scrambled control silencer RNA (siRNA, sc-41586, sc37007; Santa Cruz Biotechnology) were transfected in HepG2 and SH-SY5Y cells using Lipofectamine 2000 (ThermoScientific) reagent. Media were changed after 6 h, and cells were further incubated in growth medium for a total of 48 h. siRNA transfection efficacy was determined as > 90% of cells, using the BLOCK-iT Fluorescent Oligo (ThermoScientific).

### Gene Expression Analyses

Total RNA was isolated using RNeasy Mini kits (Qiagen) and digested with DNaseI (Promega). Reverse transcription polymerase chain reaction was performed using the SuperScriptII reverse transcription kit (ThermoScientific) and random hexamer primers (ThermoScientific) followed by IQ SYBR Green (Bio-Rad Laboratories) real-time (RT) PCR analyses on the iQ Multi-Color real time PCR detector (Bio-Rad Laboratories). Oligonucleotide sequences for the amplification of Nox4 and Nox2 and the internal standard used (acidic ribosomal protein RPLP0; NCBI Reference Sequence NM_001002.3) were

For Nox4: 5′-GACTTTACAGGTATATCCGGAGCAA-3′ and 5′-TGCAGATACACTGGACAATGTAGA-3′;
For Nox2: 5′-GCCCAAAGGTGTCCAAGCT-3′ and 5′-TCCCCAACGATGCGGATAT-3′;
For RPLP0: 5′-GTTGGTTGAAACACAGCAGCT-3′ and 5′-CAAAGGCTACCAGACGACCA-3′.

### Clinical Samples

Hepatocellular carcinoma specimen from a subject with non-alcoholic fatty liver disease was obtained during clinically indicated segmentectomy surgery from the resected part of the liver. Written informed consent to use part of the resected liver for molecular analyses was obtained from the patient.

The normal liver RNA samples used were commercially obtained (Ambion). Liver RNA was prepared from two male donors, age 69 (intracranial hemorrhage) and age 68 (intracranial hemorrhage) and one female donor age 25 (motor vehicle accident). All three were free of major infections.

### Immunoblotting

Protein extracts were prepared using 1× RIPA lysis buffer (New England Biolabs/Cell Signaling). 16 µg from each sample were mixed with 4× Laemmli buffer (Bio-Rad Laboratories), heated to 95°C for 10 min, cooled on ice and separated on a 12% SDS PAGE gel with 10 V/cm for 30 min in the stacking gel and 15 V/cm for 75 min in the separating gel. The proteins were blotted on a methanol-activated PVDF membrane with 0.45 µm pore size (Merck Millipore), at 30 V/cm electric field strength. The membrane was washed 30 min with TBS-T (25 mM Tris-HCl pH 7.4, 0.15 M NaCl, 0.5% Tween20, and 0.05% NaN_3_) before blocking unspecific binding sites with MTBS-T [5% (w/v) non-fat dry milk] for 90 min. After another washing step, the membrane was incubated with the primary antibody for 2 h. A rabbit polyclonal IgG Nox4 antibody (New England Biolabs/Cell Signaling; H-300; sc-30141; dilution 1:1,000) was used for detection of Nox4 and a rabbit monoclonal N-WASP IgG antibody (Cell Signaling; 30D10; #4848; dilution 1:2,000) for the detection of N-WASP. A rabbit polyclonal antibody to β-actin (Abcam; ab8227; dilution 1:5,000) was used for the detection of actin. A mouse monoclonal GAPDH IgG antibody (Abcam; ab9484; dilution 1:5,000) served as a loading control. Further loading controls were: cofilin using a rabbit monoclonal cofilin antibody (Cell Signaling; #5175; dilution 1:1,000), and β-actin. Following the incubation with the primary antibody the membrane was then washed with TBST-T and incubated with either goat antirabbit-HRP conjugate (Thermo Fisher Scientific; #185415; dilution 1:2,000), goat antimouse-HRP conjugate (Thermo Fisher Scientific: #31430; dilution 1:5,000), or goat antirabbit-HRP conjugate (Cell Signaling; #7074; dilution 1:2,000) as a secondary antibody. For the visualization an ECL Select Western Blotting Detection Reagent (GE Healthcare) was used according to the manufacturer’s instructions. The blot was then analyzed with the Fusion Fx7 Multi-Imagingsystem (Peqlab).

### Isolation of Microsomes

SH-SY5Y cells were trypsinized, taken up centrifuged and washed a total of three times in 10 mM HEPES buffer pH 7.7. Cells were taken up in a small volume (1.5 mL) of HEPES buffer and homogenized with a Dounce homogenizer. Sucrose was added to a final concentration of 0.25 M. Samples were centrifuged at 1,000 *g* for 10 min at 4°C. Supernatant was adjusted to 10 mL with the same buffer and centrifuged at 100,000 *g* for 30 min. The slightly brownish microsomal pellet was dissolved in 0.1 mL of RIPA buffer.

### Fluorescence Microscopy

Nox4 cDNA was cloned into pEGFP-N3 (Takara Bio Europe/Clontech) via *Kpn*I and *Not*I using the primers 5′-GGGGTACCCATGGCTGTGTCCTGGAG-3′ and 5′-AAGGAAAAAAGCGGCCGCTCAGCTGAAAGACTCTTT-3′. HepG2 and SH-SY5Y cells were grown on collagen type I-coated 22 mm round cover slips (Becton Dickinson) and transfected with 2 µg Nox4-EGFP fusion vector using Lipofectamine 2000. Cells were stained after 24 h with 100 nM MitoTracker Red CMXRos (ThermoScientific) or 1 µM ER-Tracker Blue-White DPX (ThermoScientific) for 30 min, rinsed three times in PBS (GE Healthcare), and fixed in 4% paraformaldehyd (Sigma-Aldrich) solution for 10 min at room temperature. Nuclei were stained with DAPI (Sigma-Aldrich) for 30 min. Cover slips were mounted with Fluorescent Mounting Medium (Agilent Technologies/Dako). A Zeiss LSM710 confocal microscope with an Axiocam digital camera was used for microscopic imaging.

For F-Actin staining, cells were grown overnight on collagen coated cover slips. Media was replaced with growth medium containing 50 µM DPI (diphenyleneiodonium chloride, Sigma-Aldrich) or 5 µM wiskostatin (Sigma-Aldrich) and incubated for 15 min at growth conditions. These DPI or wiskostatin treated cells were treated with 1 mM H_2_O_2_ (Sigma-Aldrich). After 15 min, cells were washed in PBS, fixed in 4% PFA, and permeabilized in 0.1% Triton X-100 (Sigma-Aldrich). F-actin filaments and cell nuclei were stained with 6 mM phalloidin-FITC conjugate (Santa Cruz Biotechnology) and 300 nM DAPI for 20 min at room temperature. After a final wash, cover slips were mounted with Fluorescent Mounting Medium. Images were analyzed using the Filaquant Software.

### Flowcytometry

HepG2 cells were trypsinized and collected in MEM Media containing 10% FBS and 50 µM DPI. After 15 min of incubation, cells were washed twice with PBS, fixed in 4% PFA (10 min at room temperature) and permeabilized with 0.1% Triton X-100 (10 min at room temperature). Cells were stained in PBS containing 6 mM phalloidin-FITC conjugate. Cellular F-actin content of treated and untreated cells was analyzed in channel FL-1 (488/530 nm) using the FACSCalibur flowcytometer and CellQuest Pro software (Becton Dickinson).

### Fluorometric Assay

SH-SY5Y and HepG2 cells were grown overnight in black Nunclon-Surface 96-well plates (Fisher Scientific). Cells were treated with 50 µM DPI or DMSO for 15 min. Subsequently, cells were rinsed with PBS and fixed in 5% PFA for 10 min at room temperature. Fixed cells were washed and permeabilized with PBS containing 0.1% Triton X-100 for 10 min. After a final rinse, cells were stained with 6 mM phalloidin-FITC conjugate, followed by RNAseA (Sigma-Aldrich) digestion and counterstaining with propidium iodide (Sigma-Aldrich). F-Actin and nuclear staining were detected with a Anthos Zenyth 3100 fluorometer (Anthos Labtec Instruments) at an excitation wavelength of 485/485 nm and an emission wavelength 535/625 nm for FITC and PI, respectively. Mean values are reported as ratio between F-actin and nuclear staining normalized to control.

### Actin Fractionation

The G-actin/F-actin *In Vivo* Assay Kit (Cytoskeleton) was used according to the manufacturer’s instructions. Untreated or hydrogen peroxide treated siRNA transfected HepG2 cells were washed in PBS and lysed in 1 mL of F-actin stabilizing buffer [50 mM PIPES pH 6.9, 50 mM NaCl, 5 mM MgCl2, 5 mM EGTA, 5% (v/v) glycerol, 0.1% (v/v) Non-idet P40, 0.1% (v/v) Triton X-100, 0.1% (v/v) Tween 20, 0.1% (v/v) 2-mercapto-ethanol, 1 mM ATP, and 1× protease Inhibitor Cocktail] for 10 min on ice. Subsequently cells were dislodged by scraping, and whole extracts were centrifuged for 1 h at 100,000 *g* in an L7-80 ultracentrifuge (Beckman Coulter, Vienna, Austria). Supernatant fractions, containing the G-actin were removed and frozen at −80°C until further use. Pellets, containing F-actin, were incubated in 1 mL of 10 µM cytochalasin D (Sigma-Aldrich) solution on ice for 1 h and vortexed every 10 min, followed by subsequent homogenization in a 1 mL glass Dounce homogenizer (Thermo Fisher Scientific/Wheaton). Photometric total protein determination was carried out using Bradford Reagent (Sigma-Aldrich) assay and a DU 640 UV/VIS spectrometer (Beckman Coulter). 5 µg protein of each fraction were loaded and separated as described in the section Immunoblotting.

Rabbit polyclonal anti-β-actin antibody (Abcam, Cambridge, UK; # ab8227; dilution 1:500) was used as the primary antibody and incubated overnight at 4°C. Goat antirabbit-HRP conjugate (Thermo Fisher Scientific/Pierce; #185415; dilution 1:2,000) was used as secondary antibody. SuperSignal West Dura Chemiluminescent Substrate (Thermo Fisher Scientific) and Kodak 2000MM Image Station were utilized to detect specific antibody binding. Integrated optical band density measurements were calculated with the Kodak1D Image Software to determine the cellular F/G-actin ratio.

### Cell Migration Assay

Cell migration activity of Nox4 or control siRNA transfected SH-SY5Y cells was studied by means of a Radius 24-well cell migration assay (Cell Biolabs) and the following media: HepG2: Eagle’s Minimum Essential Medium, low glucose (1 g/L), 10% FBS, 1% v/v penicillin/streptomycin 10,000 U/mL (Pen/Strep), and non-essential amino acids (M7145 Sigma) and SH-SY5Y: DMEM, high glucose (4.5 g/L), 10% FBS, and 1% v/v Pen/Strep. Cells were seeded into the radius 24-well plate, containing a standardized 0.68 mm hydrogel spot per well, to which cells cannot attach. Cell division (proliferation) was blocked by adding 10 µg/mL mitomycin C. After 24 h, cells reached a density of approximately 80% and subsequently the biocompatible gel was removed to start the migration into the now exposed cell-free area. After 24 h, the cells were fixed and images taken with a Nikon TMS inverted microscope and MetaView software. Area closure was quantitated using the MRI Wound Healing Tool macro for ImageJ (http://dev.mri.cnrs.fr/projects/imagej-macros/wiki/Wound_Healing_Tool).

### Statistical Analysis

Data are reported as arithmetic mean ± SD based on at least three biological replicas. Data were tested using ANOVA and Tukey *post hoc* analysis to determine significance of pairwise differences. Results were marked *p* < 0.05 (*), *p* < 0.001 (**), *p* < 0.0001 (***), and *p* < 0.00001 (****).

## Results and Discussion

The two cell lines chosen for this work are well known and widely used *in vitro* tumor models. The SH-SY5Y neuroblastoma cell line retains certain biochemical properties of dopaminergic neurons (22, 23). The HepG2 cell line retains some of the gene expression characteristics of human liver cells from which the tumor and the cell line were derived (24).

**Figure 1:**
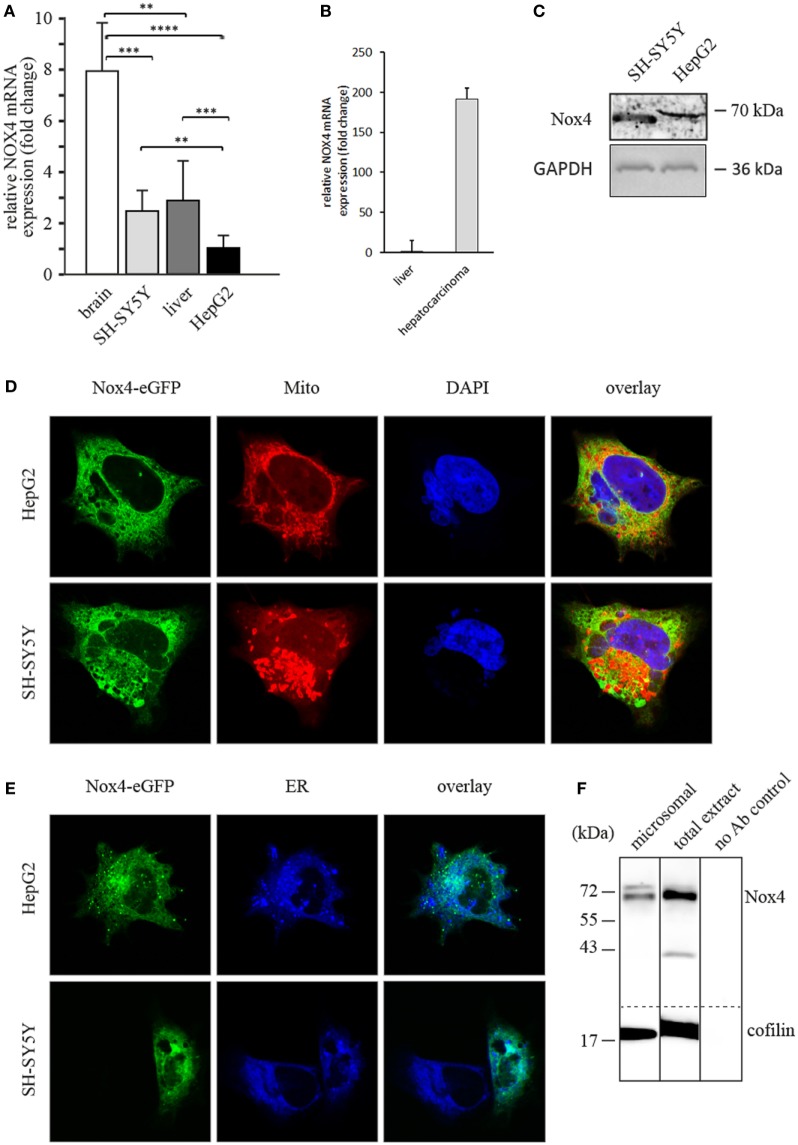
**Nox4 expression in human tissues and cultured cells and intracellular Nox4 localization in human cell lines**. **(A)** Real-time (RT) q-PCR analyses of Nox4 in human brain and liver as well as in SH-SY5Y neuroblastoma and HepG2 hepatoma cells. Values expressed as fold change relative to a housekeeping gene (ribosomal protein P0). Determination of significance as described in the Section “Materials and Methods.” **(B)** RT q-PCR analyses of Nox4 in a hepatocellular carcinoma. **(C)** Immunoblot of total protein extracts from SH-SY5Y and HepG2 cells using a polyclonal antibody against Nox4 and loading controls (GAPDH). **(D,E)** Fluorescence micrographs of HepG2 and SH-SY5Y cells transiently transfected with enhanced green fluorescence protein (eGFP) in-frame fusion construct Nox4-eGFP. DAPI, Mito or ER denotes nuclear staining with DAPI, mitochondrial staining with MitoTracker Red CMXRos, or staining of endoplasmatic reticulum with ER-Tracker Blue-White DPX, respectively. **(F)** Immunoblot of Nox4 from a total extract of SH-SY5Y cells and purified microsomes from the same extract with loading control (cofilin) and a control blot with no primary antibody.

For the purpose of the present study, we first wanted to check the amount of Nox4 expression in the tumor cell lines chosen for this work. Figure 1A shows an estimation of the amount of the Nox4 mRNA *in vivo* (human brain and liver) compared with the two cell lines. In these experiments, equal amounts of total RNA from all four sources were analyzed using the primers for Nox4 shown in the Section “Materials and Methods.” As an internal control in the same samples, the amount of the acidic ribosomal protein P0-encoding mRNA was analyzed and the values shown were normalized with respect to P0 mRNA. Clearly, the amount of Nox4 mRNA is highest in brain, but about three times smaller in the neuronal-derived cancer cell line. The amount of Nox4 mRNA in liver is smaller compared to brain, and again, the liver-derived cancer cell line contains about three times less Nox4 mRNA compared to liver. Nevertheless, the Nox4 protein is expressed in both cell lines. Next, the expression values shown in Figure 1A were compared with tumor tissue from a hepatocellullar carcinoma. In this case a 192- and 257-fold change relative to the ribosomal P0 and GAPDH, respectively, was observed (Figure 1B).

Figure 1C shows that a monoclonal antibody directed against Nox4 recognizes the protein in both cell lines. The amount of protein in the two cell lines is not dramatically different, but seems to be somewhat smaller in the liver cell line.

The next question was subcellular localization of Nox4 in the two cell lines studied here. To this end, microsomes were prepared from SH-SY5Y cells and analyzed (Figure 1F) by western blot using the same monoclonal antibody directed to Nox4 that was used before (Figure 1C). Data were normalized to cofilin in this case. The microsomes contained Nox4 protein indicating ER localization of the enzyme. These results were now confirmed by fluorescence microscopy of formaldehyde-fixed cells. Localization of Nox4 C-terminally labeled with enhanced green fluorescence protein (eGFP), mitochondria (Mitotracker Red) and nuclei (DAPI) was analyzed (Figure 1D). In a second experiment, Nox4-eGFP fluorescence was compared with an ER-specific stain (ER tracker Blue-White DPX). Figures 1D,E show that Nox4 colocalizes with the ER marker, but not with the mitochondrial or nuclear marker. We conclude that in the two cancer cell lines used here, Nox4 is located in the ER, like Yno1 in yeast (1). The role of human Nox enzymes in the ER was recently discussed with special reference to the interaction of Nox4 with protein disulfide isomerase (25).

Human Nox4 in some of the previous publications was also found in mitochondria (26) and in the nucleus (7). As mentioned before, we assume that all three localizations which have been published for Nox4 are real. Although no data exist as to the functional significance of the nuclear and mitochondrial localization, we are providing evidence here that the ER-localized Nox4 is involved in regulation of the actin cytoskeleton (see subsequently). There is evidence that the primary product of the Nox4 activity, H_2_O_2_, is exported from the ER to the cytoplasm (27) where it could then fulfill its signaling function in actin cytoskeleton remodeling.

**Figure 2:**
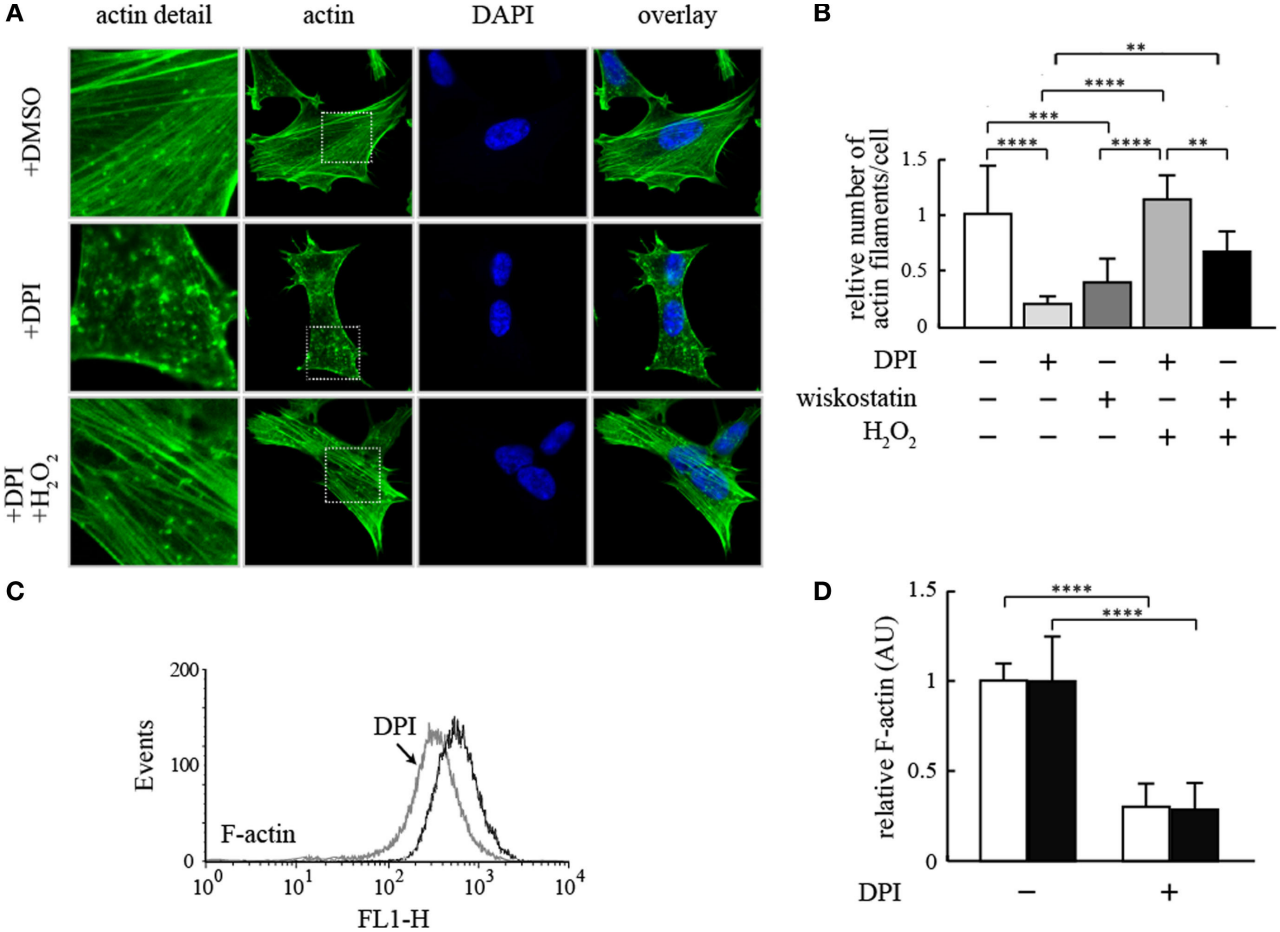
**Effect of NADPH oxidase inhibition on filamentous actin in human neuroblastoma cells**. **(A)** Representative fluorescence micrographs of SH-SY5Y cells stained with phalloidin-FITC and DAPI. Cells were treated with DMSO, 50 µM DPI or 50 µM DPI, and 1 mM H_2_O_2_ for 15 min. Cells were washed, fixed and actin filaments and cell nuclei were stained with phalloidin-FITC and DAPI, respectively. **(B)** Actin filament number of 100 cells treated as in **(A)** or with 5 µM wiskostatin was analyzed with the software FilaQuant from fluorescence micrographs and expressed relative to the number found in untreated cells without DPI, wiskostatin, or H_2_O_2_. Results are mean ± SD and were marked for significance as described in the Section “Materials and Methods.” **(C)** Detached HepG2 cells were treated with 50 µM DPI or DMSO, respectively. Cells were stained with phalloidin-FITC prior to FACS analysis for F-actin content. Results of one representative experiment are shown. **(D)** HepG2 and SH-SY5Y cells were treated with 50 µM DPI or DMSO for 15 min, respectively. Cells were stained with phalloidin-FITC and treated with RNAse A prior to counterstaining with propidium iodide. F-actin and nuclear staining was determined with a fluorometric assay. F-actin content is shown as normalized to nuclear staining intensity. Open bars denote HepG2 cells, whereas filled bars denote SH-SY5Y cells. Results are mean ± SD and were marked for significance as described in the Section “Materials and Methods.”

**Figure 3:**
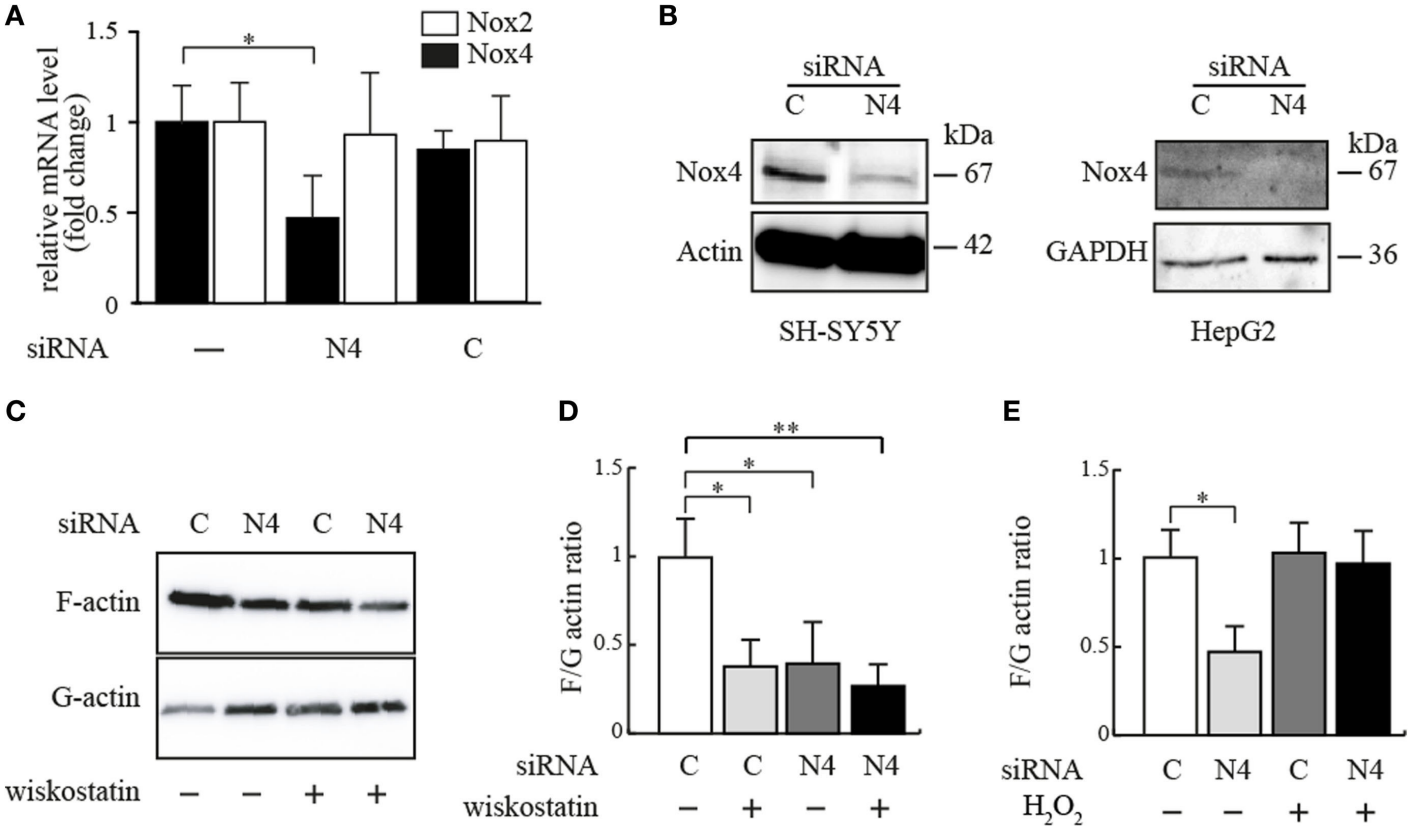
**Effect of siRNA-mediated Nox4 knockdown on cellular F- and G-actin content**. **(A)** Nox4 mRNA expression was determined using real-time PCR in SH-SY5Y cells cultured for 48 h in the presence of either Nox4 or scrambled siRNAs. Values expressed as fold change relative to a housekeeping gene (ribosomal protein P0). Results are mean ± SD and were marked for significance as described in the Section “Materials and Methods.” **(B)** Anti-Nox4 immunoblot analysis of total protein extracts isolated from Nox4 siRNA or control siRNA-treated SH-SY5Y and HepG2 cells, transiently transfected for 48 h. As loading controls, beta-actin and GAPDH immunoblots were used. **(C)** Representative immunoblot of actin after fractionation by ultracentrifugation to discriminate cellular F-actin content from G-actin in homogenates from HepG2 cells treated with Nox4 or control siRNA for 48 h in the presence or absence of 5 µM wiskostatin for 15 min. **(D)** F/G ratios calculated from densitometrical analysis of three independent experiments as in **(C)**, normalized to cells receiving control siRNA only. Results are mean ± SD and were marked for significance as described in the Section “Materials and Methods.” **(E)** F/G actin ratios calculated from densitometric analysis of immunoblots for actin after fractionation of F-actin and G-actin in homogenates from HepG2 cells treated with Nox4 or control siRNA for 48 h in the presence or absence of 1 mM hydrogen peroxide for 15 min. Results are mean ± SD and were marked for significance as described in the Section “Materials and Methods.”

Next, we studied a possible connection between Nox4 activity and regulation of the actin cytoskeleton by staining of F-actin (after formaldehyde fixation) in growing cells in the absence and in the presence of inhibitors. As shown in Figure 2A, blocking NADPH oxidase activity with the unspecific inhibitor, DPI, led to a marked change in the appearance of the F-actin morphology of the SH-SY5Y cells. The normal network of F-actin fibers largely disappeared concomitantly with the appearance of granular F-actin aggregates. Interestingly, this effect was reversed by adding a low, non-toxic concentration of H_2_O_2_ to the cells (Figure 2A). To quantify this effect, we counted the number of actin microfilaments per cell (Figure 2B) leading to an estimate of about 25% remaining actin microfilaments. This effect was mostly due to inhibition of Nox4, which is the predominant NADPH oxidase of the neuronal cell line used (6). This fact was also shown by specifically inhibiting Nox4 by siRNA techniques (Figures 3A,B). A similar effect was achieved by treating the cells with wiskostatin (Figure 2B), an inhibitor of actin filament nucleation, especially at the branching points of the actin microfilament network, which is needed for the dynamics of the actin cytoskeleton in growing cells, in particular at the polarized moving edge of moving cells, and in the process of endocytosis. The effect of wiskostatin was partially reversible by H_2_O_2_ (Figure 2B). A similar effect was shown previously for the yeast NADPH oxidase, Yno1 (1). Taken together, the results shown so far point to a possible explanation for the mechanism of action of Nox4 in the two tumor cell lines: Nox4 seems to create a signaling substance, which is probably H_2_O_2_ (because the lack of Nox4 can be compensated by H_2_O_2_) and needed for regulation of the branching mechanism of the dynamic actin cytoskeleton of living cells.

F-actin is not only changing its morphological appearance but also in part converted into the monomeric G-actin which is no longer stainable with rhodamine-phalloidin. After staining for F-actin, the detached cells of the HepG2 culture were analyzed by FACS (Figure 2C), showing an appreciable loss of F-actin after DPI inhibition of NADPH oxidase. In a similar experiment, F-actin rhodamine-phalloidin fluorescence was quantitatively determined in both the HepG2 and SH-SY5Y cells with and without DPI, and the fluorescence was normalized to the fluorescence intensity of the propidium iodide stained nuclei of the cells. In both cell types, F-actin content was decreasing to about 25% of the undisturbed value after DPI inhibition (Figure 2D).

The effect of the Nox4-specific siRNA on Nox4 transcript levels in SH-SY5Y cells is shown in Figure 3A, using RT-PCR. Expressing the siRNA construct *in vivo* leads to a decrease of the Nox4 mRNA to about 45% of the normal undisturbed value. The construct has no influence on Nox2 expression, showing the specificity of the siRNA construct and there is no effect of the “scrambled” siRNA construct on the transcript levels. The decrease in mRNA is reflected by the amount of protein as determined in western blots (Figure 3B). It is important to note that the western blot results shown in Figures 1B and 3B both show the full length protein, not one of the substantially smaller splicing isoforms. As shown in Figure 3B, expression of Nox4 at the protein level is strongly diminished in both cell lines by the siRNA used.

A commercial kit was now used to separate and quantitate F-actin and the monomeric form (G-actin) from the cells and to study the simultaneous action of Nox4 siRNA and wiskostatin. In undisturbed cells, there is a large majority of F-actin (Figure 3C, first lane). After wiskostatin treatment or in cells treated with Nox4 siRNA, the F/G-actin ratio is about 1 indicating approximately equal amounts of the two forms of actin (Figure 3C, lanes 2–4). Importantly, the combination of Nox4 siRNA and wiskostatin leads to a F/G ratio which is not different from either of the two inhibitors alone (Figure 3D). There is no additive effect of siRNA and wiskostatin. The standard (but cautious) interpretation of this finding is that the two inhibitors act on the same process in the cell, namely actin filament nucleation and branching, perhaps in a sequential manner. The shift from F-actin to G-actin is reversible by H_2_O_2_ (Figure 3E). Taken together, these findings mean that Nox4 exerts an activity directed to growth and/or branching of actin filaments, which is very probably mediated by the second messenger produced by Nox4, hydrogen peroxide.

**Figure 4:**
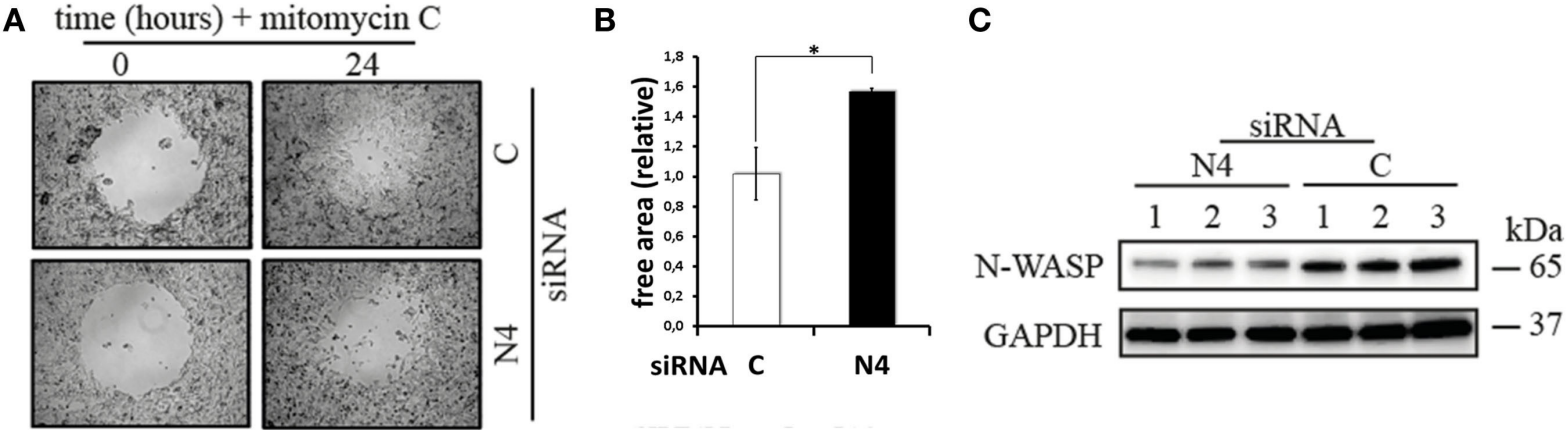
**Cell migration assay**. **(A)** SH-SY5Y cells either transfected with Nox4 or scrambled siRNA were grown to 80% confluency in a 24-well plate containing a hydrogel spot (time point 0 h). After removing of the spot the cells transfected with scrambled siRNA but not the Nox4 siRNA started to migrate into the cell free area (time point 24 h). **(B)** The remaining free area in the open spots was determined as described in the Section “Materials and Methods.” Results are mean ± SD and were marked for significance as described in the Section “Materials and Methods.” **(C)** The western blot for N-WASP normalized to GAPDH indicates downregulation of N-WASP when Nox4 expression was suppressed in three independent homogenates from hepatoma cells. Equal amounts of protein were loaded onto the SDS-PAGE.

The results discussed so far prompted us to investigate the influence of Nox4 inhibition and the concomitant shift in the actin cytoskeleton on mobility of the two tumor cell lines in so-called “scratch” assays (Figure 4). Inhibiting Nox4 in the SH-SY5Y cells led to a significant loss of mobility of the cells leading to a larger area not covered by migrating cells (Figures 4A,B). Possibly, this finding supports a role for Nox4 in regulating actin cytoskeleton dynamics, which is needed in the process of metastasis. This finding would mean that in neuroblastoma, the tumor from which the SH-SY5Y cell line is derived, Nox4 is a drug target worth considering. However, the effect observed is not a general one, as in the HepG2 cell line transfection with the Nox4 siRNA did not inhibit cell migration (data not shown). This is surprising as the level of Nox4 protein is very low under these conditions (Figure 3B) and possibly indicates that the regulation of the actin cytoskeleton nucleation and branching as well as the regulation of cell migration must be in part different in the two cell lines.

Finally, in Figure 4C, we show that downregulating Nox4 mRNA by siRNA leads to lowering of N-WASP, the probable drug target of wiskostatin which is mechanistically directly involved in the branching process of actin microfilaments. How can this finding be explained? We think that a direct feedback circuit probably exists which downregulates the amount of N-WASP protein in times when it is not used as a signaling target.

## Conclusion

The experimental results presented and discussed herein lead to a still hypothetical but consistent picture of the role of the NADPH oxidase, Nox4, in cellular growth in normal and tumor cells. Nox4 which in the two tumor cell lines studied here is a NADPH oxidase of the ER, directly (without the help of superoxide dismutase) produces H_2_O_2_ as a growth-related signaling substance. Attenuation of this signaling module leads to characteristic changes in the actin cytoskeleton, like decomposition of actin microfilaments. A complete loss of Nox4 in HeLa cells leads to a much more drastic effect of loss of both proliferation and cell migration (28). However, these knock-out cells are still viable. Presently unknown is the recipient of H_2_O_2_ which presumably transmits the signal to the complicated machinery which regulates the actin cytoskeleton, in particular actin nucleation and branching (29). The point of attack of the signal is near the point where wiskostatin acts (i.e., F-actin branching and nucleation), as judged by the combination experiments with Nox4 inhibition and wiskostatin inhibition of N-WASP. As this inhibition blocks cell mobility and, therefore probably metastasis, in one of the two cell lines studied here, we suggest to study Nox4 as a drug target in cancer therapy. This must be done with great care, as the possible unwanted side effects of such treatment are unknown and some published experiments (30) not only point to a function of Nox4 in tumor growth and metastasis, but also to a function of Nox4 in the process of apoptosis, which should not be blocked in tumor therapy.

## Author Contributions

MB, TF, and MR wrote the manuscript. MB, MR, EH-B, EA, and TF designed experiments. MR, TF, SA, JB, MKS, HB-K, RG, JC, KR, and MS performed experiments.

## Conflict of Interest Statement

The authors declare that the research was conducted in the absence of any commercial or financial relationships that could be construed as a potential conflict of interest.
